# Analysis of Mesquite (*Prosopis juliflora*) Protein Concentrate for Possible Use as Supplementary Protein

**DOI:** 10.1155/2022/7621818

**Published:** 2022-02-24

**Authors:** José Jaimes-Morales, Yesid A. Marrugo-Ligardo, Diofanor Acevedo-Correa

**Affiliations:** ^1^Grupo de Investigación en Medio Ambiente, Alimentos y Salud, Universidad de Cartagena, Campus Zaragocilla, Cartagena de Indias 130015, Colombia; ^2^Grupo de Investigación en Innovación y Desarrollo Agropecuario y Agroindustrial (IIDAA), Universidad de Cartagena, Campus Piedra de Bolívar, Cartagena de Indias 130015, Colombia

## Abstract

Vulnerable populations in developing countries need new protein sources, such as protein concentrates from accessible sources at low economic costs. The main objective of this study was at evaluating the nutritional quality of the protein concentrate of the legume mesquite (*Prosopis juliflora*), compared with the protein values of other legumes described in literature. For this purpose, flour and protein concentrates from mesquite were obtained, along with their chemical composition. Amino acid profiling was performed by high-performance liquid chromatography (HPLC). Protein quality index evaluation tests were also performed on preschool children and adults. The protein content of mesquite was found to be 68%. However, mesquite covers the requirements of essential amino acids, surpassing 31% of the protein required in adults, except for cysteine sulfur amino acids and aromatic amino acids. In other age groups such as children, mesquite had a high content of histidine, which is necessary and considered essential during infant development. According to the above, mesquite could be used as an alternative protein to produce food with high nutritional content.

## 1. Introduction

The food security of the population is still affected by malnutrition in many regions of the world, especially in developing countries in Africa, Asia, and Latin America [1–4]. In these areas of the planet, the lack of access to food is growing at an accelerated rate, with the most vulnerable populations requiring mainly protein sources. In this context, dietary protein requirements are affected by the high cost of traditional protein sources, due to the high import rates of legumes such as soybeans, and the lack of native vegetable production [5, 6].

Due to the crisis caused by the COVID-19 pandemic, the current and future states of sustainable food security have worsened. Rising food prices, disruptions in supply chains, and limited access to food [7–9] have exacerbated these issues even more. Alternative food sources need to be available and accessible to overcome the issues related to the lack of access to foods of high nutritional value. Some of the most promising solutions are those that can be obtained from plant sources, such as cereals, fruits, legumes, and vegetables. Many of the food sources currently being studied as unique sustenance alternatives are deficient in some vitamins; however, they can be complemented with the consumption of other species, which must be subjected to processing to be digestible and absorbable by the human metabolism [10–12].

Balance between dietary supply and protein needs is also important, as it is vital for maintaining the health and well-being of the population. To this end, alternative sources of protein should be explored in areas that require it, investigating the quality of proteins from native plant species that meet the required nutritional value and thus transition to more sustainable plant diets [5, 13]. Legumes fit into this type of diet since they have beneficial nutritional properties in terms of their high protein content and good amino acid profile, thus being an important source for human nutrition, especially in low-income communities. In their composition, their protein values range depending on the species from 17% to 40%, with even higher amounts of carbohydrates, and variable oil content, normally from 1% to 6%. Soybeans, beans, chickpeas, and lentils are the most relevant in terms of production volumes, market value, and nutritional value [14, 15]. Regarding the protein potential of their seeds, legumes have gained importance in the food industry as ingredients in dietary nutritional formulas, meat alternative products [13, 14], animal feed, vegetable oil, and protein concentrate [16, 17].

The *Prosopis* genus comprises 44 species of nitrogen-fixing trees that grow mainly in arid or semiarid regions of the Americas. Prosopis pods are sweet fruits consisting of 70%–75% pericarp (epicarp, mesocarp, and endocarp) and 25%–30% seeds (episperm, endosperm, and cotyledons). The whole ripe pods of the mesquite are ground to produce flour, known as mesquite or carob flour, characterized by its brown color, and coffee-like aroma [18].

The nutrient content and protein digestibility of mesquite flour varies according to its variant. In addition, mesquite flour has been reported to be a good source of lysine, sulfur-containing type of amino acids (Glu, Arg, Asp, and Leu), and total phenolic compounds with increased antioxidant capacity [19].

The ripe fruit of this legume is a seed pod with high nutritional value. The seeds are found in the wild and have low commercial value. It is a legume of the bean, pea, caraota, and quinchoncho family, *Mimosaceae* [20–23]. The protein of mesquite constitutes 60% of the weight of the seeds, but for animals to benefit from it, the pods and seeds must be crushed; otherwise, they pass through the digestive tract without being digested. Crushing is difficult because of the presence of a thick pulp surrounding the seed [24, 25].

The nutritional, physicochemical, and biological characteristics of mesquite promote it as a new source of proteins, which make it possible to increase the supply and make high-protein foods available to the population at more affordable prices [19, 26, 27]. The use of protein concentrates from native plant species such as mesquite for possible addition as a nutritional ingredient is an opportunity to provide the community with a food alternative of great impact on nutritional problems and promote the production of this native plant that needs more technical cultivation methods.

Considering the information available on mesquite and its possible use as a protein source, the objective of this study was to evaluate the quality of protein present in mesquite concentrate as a food with high nutritional content, comparing it with that of the soybean, which is a well-known legume used by the food industry.

## 2. Materials and Methods

### 2.1. Sample Collection

**Figure 1:**
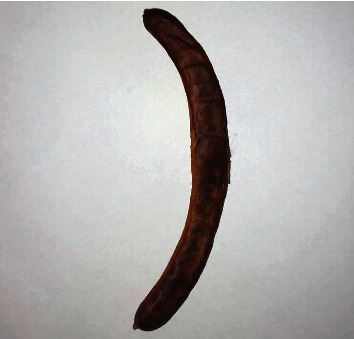
Mesquite used in this work.

800 g of mesquite seeds (see Figure 1) was collected and studied in good physical conditions of development, state of maturity, and phytosanitary hygienic conditions. The mesquite was collected from Cerro de la Popa, located in the city of Cartagena de Indias, Colombia.

### 2.2. Obtaining Integral Concentrate from Mesquite Seeds

The procedure proposed by Jaimes-Morales et al. [24] was followed to obtain whole mesquite flour. The concentrate was made from pods in good physical and hygienic phytosanitary conditions, then milled, and passed through 1 mm^2^ no. 2 and no. 3 filters, thus obtaining fine integral concentrate, free of unwanted residues.

### 2.3. Obtaining Mesquite Protein Concentrates

To obtain the mesquite protein concentrate, the Soxhlet method was used under moderate thermal conditions not exceeding 40°C, in an attempt to preserve the nutritional properties of the proteins. The defatted flour was subjected to flour-water suspensions in a 1 : 8 ratio. In the extraction of soluble carbohydrates and mineral salts, the pH was adjusted to 8 using NaOH 1N. These suspensions were centrifuged at 300 rpm and 40°C for 30 min. At the end of the extraction period, the mixture was left to rest, removing the supernatant and the amylaceous residue. Then, the procedure was repeated. The supernatants of the washes and the protein extract were collected in a 50 mL beaker. The extract was acidified with 1N HCl until the precipitation point of most of the proteins was reached at pH 5.5. The protein extract was kept at 37°C and then subjected to shaking at 300 rpm for 30 min through centrifugation. Finally, the proteins were separated from the serum by decantation and washed with water under constant mixing. To obtain the protein coagulum, it was subjected to decantation and vacuum drying.

### 2.4. Comparison of Chemical Composition of Mesquite

The chemical components of the protein concentrates were determined as follows. For the mesquite concentrate, the methodology of Sarmiento et al. [26] and the AOAC (2003) [28] were used. The factors determined were moisture, fiber, ash, fat, and protein. These values were added and subtracted from 100, and the difference was taken as the carbohydrate content.

### 2.5. Determination of Essential Amino Acids in Mesquite

The determination of amino acids was made by high-performance liquid chromatography (HPLC) [29] using a BAS chromatograph (California, USA) with a Water 474 Fluorescence Detector. The fluorescence detection was performed using an excitation wavelength of 340 nm and an emission wavelength of 460 nm.

### 2.6. Chemical Computation of Amino Acids to Mesquite

To determine the chemical count of the mesquite protein concentrate, the method proposed by Marrugo et al. [30, 31] was followed, which consisted of dividing the number of essential amino acids present in the protein concentrates by the number of essential amino acids in the standard protein and multiplying this value by one hundred.

### 2.7. Evaluation of the Protein Quality Index of Mesquite

This value was calculated for the concentrates under study by applying the method proposed by Frota et al. [32] and Sarmiento et al. [26]. This consisted of dividing the protein requirement recommended by the FAO by the requirement of the most limiting amino acid of the protein under study. Similarly, for the calculation of the protein requirement recommended by the FAO at a given age, the methods proposed by the aforementioned authors were used, where the requirement of the most limiting amino acid indicated in the ideal protein proposed by the FAO/WHO was divided by the amount of limiting amino acid of the protein being evaluated [33, 34].

### 2.8. Data Processing and Analysis

Data was collected from bibliographic references of the research background and laboratory data from the physicochemical and biochemical analysis of the concentrates, and the results were tabulated in Microsoft Excel 2010 spreadsheets. The calculations were carried out in triplicate and the results were expressed as the mean ± standard deviation.

## 3. Results and Discussion

### 3.1. Chemical Composition of Mesquite

Table 1 shows crude fiber, ash, moisture, and carbohydrate contents of mesquite. According to the data obtained in Table 1, the protein content of mesquite was like those of other legumes such as soybeans [33], which demonstrates the potential of this legume in the preparation of plant-based foods with high protein content or as a substitute for meat products. It is also important to highlight the importance of developing research where the biological value and the type of proteins present in it are indicated [26].

The protein values (in mesquite concentrate) were higher than those reported by Ugwuona and Suwaba [31] for protein concentrates of sword beans (*Canavalia ensiformis*) with a 49.5% protein content. On the other hand, lower protein values were recorded in mesquite concentrate than those reported by Ghribi et al. [35] for chickpea (*Cicer arietinum* L.) concentrates. Regarding the fiber content, mesquite concentrate showed substantially lower values than soybean concentrate. These results for mesquite were like those presented by Betancur-Ancona et al. [36], who reported the same value in ancho beans (*Phaseolus lunatus*). The fat contents of protein concentrates for both mesquite and soybean were low, compared to those of the studies conducted by Cruz-Gracida [37] for carob bean (*Prosopis laevigata)* concentrate and by Ghribi et al. [35] for chickpea concentrate.

Although the digestibility of mesquite concentrate was not measured in this study, there are studies such as that of Mamone et al. [25] which found that the germ meal of this legume is highly digestible in the gastrointestinal tract, releasing significant amounts of free amino acids.

### 3.2. Amino Acid Profile of Mesquite

The amino acid composition of mesquite proteins is shown in Table 2. Leucine, methionine + cysteine, phenylalanine + tyrosine, tryptophan, and histidine contents had the highest values. Threonine was not detected in the concentrate.

The amino acid profile showed evident nutritional potential of mesquite. Comparing the data obtained in our study with those reported by Deak et al. [33], the amino acids leucine, methionine + cysteine, phenylalanine + tyrosine, tryptophan, valine, and histidine are found in higher proportions in mesquite compared to soybean, with differences of 26.50 mg/100 g, 41.40 mg/100 g, 20.30 mg/100 g, 14.30 mg/100 g, 1.20 mg/100 g, and 39.20 mg/100 g, respectively. Concerning amino acids such as isoleucine, lysine, and threonine, their content was higher in soybeans. According to our data, mesquite concentrate did not contain threonine, so it is necessary to mix different legumes such as soybeans to obtain concentrate with complete protein content.

Compared to other legumes, e.g., peas [38], the amino acid content of mesquite was higher in those analyzed in this study and the protein content was like those of concentrates such as peas. However, care must be taken with these comparisons, as the amino acid and protein content of legumes may change according to the variety [39].

It should be noted that histidine, one of the amino acids with the highest concentration in mesquite concentrate, is considered essential during infancy. This leads us to consider the use of concentrate as a possible therapeutic food [40].

Interesting aspects were found in this research regarding the essential amino acid content of mesquite and soy protein concentrates concerning the amino acid requirements in different age groups according to FAO/WHO/UN. In the case of isoleucine, mesquite had a slightly lower content than that found in soy concentrate and was like what is required by infants (4.6 mg/100 g of protein). But it was higher than what is required by other age groups such as children from 2 to 5 years old, school children from 10 to 12 years old, and adults, with percentage differences of 39%, 39%, and 72%, respectively.

The leucine content found in mesquite concentrate was 4 times higher than that reported for soy concentrate. Regarding the age groups of infants, children, school children, and adults, it was found that the contents for this amino acid were up to 3, 5, 12, and 18 times higher, respectively, than what is recommended by FAO. This means that the daily requirements of this amino acid would be completely covered by using mesquite concentrate in starchy products for mass consumption. This trend was similar for amino acids such as methionine, tryptophan, and histidine since the standard requirements reported by FAO for these three amino acids range for infants from 17 mg/100 g to 42 mg/100 g of protein, for children from 11 mg/100 g to 25 mg/100 g of protein, for school children from 9 mg/100 g to 22 mg/100 g of protein, and for adults from 5 mg/100 g to 16 mg/100 g of protein.

### 3.3. Chemical Computation of Essential Amino Acids in Mesquite

Chemical computations of mesquite protein concentrate for the preschool age study from 2 to 5 years old are shown in Table 3. This calculation is a parameter for measuring the quality of protein in the diet, based on the composition of essential amino acids of the concentrates under study (Table 2) and the standard or ideal protein according to FAO/WHO/UN. The values established for each essential amino acid were isoleucine (2.8 ± 0.0), leucine (6.6 ± 0.0), lysine (5.8 ± 0.0), methionine + cysteine (2.5 ± 0.0), phenylalanine + tyrosine (6.3 ± 0.0), threonine (3.4 ± 0.0), tryptophan (1.1 ± 0.0), and valine (3.5 ± 0.0). According to the results obtained, those amino acids that are found in greater proportion or in a relationship of inverse proportion between the value of the amino acid in the study concentrate and the protein standard would be better. According to the above, methionine + cysteine and tryptophan were the amino acids with dispositions to supply the requirements for the ideal protein proposed by FAO/WHO/UN.

The chemical score is an index of protein quality, which is measured inversely to the chemical computation, since the analysis is based on the relationship between the ideal proteins for comparison, in this case, the preschool protein and the studied protein. In this work, the chemical scores for the mesquite protein concentrate studied were inverse to the chemical computation in its determination ratio.

The mesquite protein concentrate satisfied the essential amino acid requirements for this group according to the FAO/WHO/UN ideal protein. In general, mesquite could be useful in the design of foods with high protein content and, in turn, could supply the amino acid needs of other foods. However, due to its low content of some amino acids that are present in other legumes such as soybeans, it would be ideal to make mixtures of these types of vegetables to ultimately obtain foods with high nutritional content, especially protein.

Sarmiento et al. [26] and Díaz-Batalla et al. [41] functionally characterized mesquite flour to include it in a food matrix as a protein extender. Similarly, the functional properties of mesquite protein concentrate obtained by different methods for possible use as a food ingredient reported by Jaimes-Morales [24] can be evidenced.

### 3.4. Evaluation of the Protein Quality Index of Concentrate of Mesquite

Table 4 shows the protein quality index and protein scores of mesquite in preschool children and adults, respectively. For their determination, a relation with the amino acid and protein requirements according to FAO/WHO recommendations had to be made. The established values were for preschoolers between 2 and 5 years of age in 1 g of ideal protein and whose amino acid values in mg were isoleucine (28 mg), leucine (66 mg), lysine (58 mg), methionine + cysteine (25 mg), phenylalanine + tyrosine (63 mg), threonine (34 mg), tryptophan (11 mg), valine (35 mg), and histidine (19 mg). For adults, the ideal protein value was 0.75 g, whose amino acid values were isoleucine (13 mg), leucine (19 mg), lysine (16 mg), methionine + cysteine (17 mg), phenylalanine + tyrosine (19 mg), threonine (9 mg), tryptophan (5 mg), valine (13 mg), and histidine (16 mg).

The protein quality index was calculated as the ratio of the protein requirement of the FAO/WHO/UN recommended standard for an age group in g/kg/d divided by the score of the most limiting amino acid value of the required protein intake of the studied protein multiplied by 100%. The protein intake score of the amino acid requirement of the studied protein was obtained by dividing the mg of amino acid of the FAO ideal protein by the mg of the respective amino acid of the studied protein.

The nutritional quality of a protein depends primarily on its ability to meet essential amino acid requirements. Nitrogen and amino acid demands are the most logical measures for predicting the quality of a protein. Mesquite met the amino acid requirements for adults except for the sulfur amino acids for cysteine and aromatic amino acids. When calculating the protein quality index of the concentrates for preschoolers, it was observed that the protein of the soy concentrate had a higher protein quality index compared to the protein of the mesquite concentrate. The values also indicated that both concentrates exceeded the amino acid requirements by more than 100%, according to the FAO/WHO ideal protein. The valine content found in mesquite was 63 mg/100 g of protein, which was 19% higher than that reported for soy concentrate. Likewise, it was 11% higher than that reported in the recommended daily requirement for infants and 44% higher than the recommended daily requirement for children. The content of this amino acid in mesquite was up to 3 and 4 times higher [24, 36] for school children and adults.

When calculating the protein quality index of the concentrates studied for adults, it was observed that the mesquite concentrate was 208%. These values also indicated that both concentrates exceeded the amino acid requirements by more than 100%, according to the FAO/WHO/UNU ideal protein.

## 4. Conclusions

According to our results, mesquite should be considered as a suitable alternative in the development of foods with high nutritional quality. Also, from an economic perspective, our study considered the use of legumes such as mesquite in the development of sustainable agricultural productivity in production areas and where there are possibly vulnerable populations in a state of malnutrition due to poor access to food. However, it is important to highlight the need to test the digestibility of mesquite protein to assess its protein quality more accurately.

The protein concentrate of mesquite, due to its nutritional characteristics in terms of protein content, available protein, chemical computation, and protein quality index, could offer possible nutritional enrichment when incorporated in certain types of food, since it covers the requirements of essential amino acids for adults (exceeding 31% of the protein required for this age group) and it fulfills with equal conditions the requirements of other amino acids in the other age groups.

## Figures and Tables

**Table 1:** Chemical composition of mesquite.

Physicochemical properties	Composition (%)
Total protein	67.9 ± 0.3
Fat	0.8 ± 0.1
Crude fiber	1.9 ± 0.4
Ash	4.3 ± 0.1
Moisture	0.9 ± 0.4
Carbohydrates	24.2 ± 1.1

Values are presented as mean ± standard deviation (*n* = 3 × 2).

**Table 2:** Essential amino acid profile of mesquite concentrate.

Essential amino acid	Content (mg/100 g of protein)
Isoleucine	4.6 ± 2.1
Leucine	34.4 ± 0.7
Lysine	4.4 ± 1.7
Methionine + cysteine	47.0 ± 0.8
Phenylalanine + tyrosine	38.1 ± 0.9
Threonine	Not detected
Tryptophan	15.9 ± 1.4
Valine	6.2 ± 0.8
Histidine	45.6 ± 0.5

Values are presented as mean ± standard deviation (*n* = 3 × 2). Analysis was performed by HPLC (high-performance liquid chromatography).

**Table 3:** Chemical computation and score on the composition of essential amino acids in mesquite protein concentrate.

Essential amino acid	Chemical computation	Chemical score
Isoleucine	1.6 ± 0.8	0.6 ± 0.1
Leucine	5.2 ± 0.1	0.2 ± 0.1
Lysine	0.8 ± 0.3	1.3 ± 0.0
Methionine + cysteine	18.8 ± 0.3	0.1 ± 0.0
Phenylalanine + tyrosine	6.0 ± 0.1	0.2 ± 0.5
Threonine	Not detected	Not detected
Tryptophan	14.4 ± 1.4	0.1 ± 0.0
Valine	1.8 ± 0.3	0.6 ± 0.1

Values are presented as mean ± standard deviation (*n* = 3 × 2).

**Table 4:** Protein quality index and score of mesquite concentrate.

Essential amino acid	Amino acid composition (mg/g)	Protein intake score of child requirements (g/kg/d)	Protein intake score of adult requirements (g/kg/d)
Isoleucine	46	0.61	0.28
Leucine	344	0.19	0.06
Lysine	44	1.32	0.36
Methionine + cysteine	470	0.05	0.04
Phenylalanine + tyrosine	381	0.16	0.05
Threonine	Not detected	Not detected	Not detected
Tryptophan	159	0.07	0.03
Valine	62	0.56	0.21
Histidine	456	0.04	0.04

## Data Availability

The data used to support the findings of this study are available from the corresponding author upon request.
